# Supplementary material for: Overcoming barriers to the registration of new plant varieties under the DUS system
Source: Commun Biol. 2021 Mar 8;4:302. doi: 10.1038/s42003-021-01840-9 (PMC7940638; doi:10.1038/s42003-021-01840-9)
Supplement: Supplementary file 2 — Supplementary Information [file 42003_2021_1840_MOESM2_ESM.pdf]

Supplementary Materials

Overcoming barriers to the registration of new plant varieties under the DUS system.

Chin Jian Yang

Joanne Russell

Luke Ramsay

William Thomas

Wayne Powell

Ian Mackay

Supplementary Table 1. Genetic variances, phenotypic variances and heritabilities for 28 DUS traits. Estimates of genetic variance (V_G), phenotypic variance (V_P) and heritabilities (h^2) are shown along with their standard errors in parentheses.

Trait	V_G			V_P			h^2		
	Combined	Spring	Winter	Combined	Spring	Winter	Combined	Spring	Winter
1	0.22 (0.02)	0.01 (0.00)	0.31 (0.05)	0.28 (0.02)	0.07 (0.01)	0.40 (0.04)	0.78 (0.04)	0.16 (0.06)	0.79 (0.06)
2	0.29 (0.06)	0.19 (0.06)	0.25 (0.08)	1.16 (0.06)	1.09 (0.08)	1.04 (0.08)	0.25 (0.05)	0.17 (0.06)	0.24 (0.07)
3	1.86 (0.20)	NA	2.96 (0.47)	2.48 (0.16)	NA	4.29 (0.37)	0.75 (0.04)	NA	0.69 (0.07)
4	1.94 (0.23)	0.19 (0.06)	3.01 (0.49)	2.62 (0.18)	1.04 (0.08)	3.59 (0.37)	0.74 (0.05)	0.19 (0.06)	0.84 (0.08)
5	0.91 (0.40)	0.79 (0.49)	0.68 (0.41)	3.21 (0.37)	2.83 (0.53)	2.69 (0.44)	0.28 (0.13)	0.28 (0.19)	0.25 (0.16)
6	0.12 (0.04)	0.03 (0.03)	0.14 (0.07)	1.04 (0.06)	0.75 (0.06)	1.34 (0.11)	0.12 (0.04)	0.05 (0.03)	0.10 (0.05)
7	0.53 (0.11)	0.28 (0.09)	0.56 (0.16)	1.88 (0.10)	1.41 (0.11)	2.16 (0.17)	0.28 (0.05)	0.20 (0.06)	0.26 (0.07)
8	1.46 (0.18)	0.10 (0.05)	2.29 (0.38)	2.17 (0.15)	1.02 (0.08)	2.78 (0.29)	0.67 (0.05)	0.09 (0.04)	0.83 (0.08)
9	0.65 (0.10)	0.52 (0.10)	0.54 (0.14)	1.55 (0.09)	1.14 (0.09)	1.66 (0.13)	0.42 (0.05)	0.45 (0.07)	0.33 (0.08)
10	0.47 (0.10)	0.36 (0.10)	0.35 (0.13)	1.86 (0.10)	1.39 (0.10)	2.12 (0.17)	0.25 (0.05)	0.26 (0.07)	0.17 (0.06)
11	0.27 (0.07)	0.18 (0.07)	0.23 (0.10)	1.58 (0.09)	1.42 (0.11)	1.65 (0.13)	0.17 (0.04)	0.13 (0.05)	0.14 (0.06)
12	0.01 (0.00)	NA	0.01 (0.00)	0.01 (0.00)	NA	0.01 (0.00)	1.00 (0.01)	NA	1.00 (0.03)
13	0.08 (0.03)	0.03 (0.03)	0.07 (0.04)	0.85 (0.05)	0.86 (0.07)	0.81 (0.07)	0.10 (0.04)	0.04 (0.03)	0.09 (0.05)
14	0.28 (0.06)	0.15 (0.06)	0.29 (0.09)	1.23 (0.07)	1.12 (0.08)	1.24 (0.10)	0.23 (0.05)	0.14 (0.05)	0.24 (0.07)
15	0.12 (0.03)	0.04 (0.03)	0.17 (0.05)	0.69 (0.04)	0.74 (0.06)	0.58 (0.05)	0.18 (0.05)	0.05 (0.04)	0.29 (0.08)
16	0.14 (0.04)	0.08 (0.03)	0.11 (0.05)	0.80 (0.04)	0.54 (0.04)	1.01 (0.08)	0.18 (0.04)	0.15 (0.05)	0.11 (0.05)
17	0.23 (0.04)	0.18 (0.04)	0.20 (0.05)	0.68 (0.04)	0.57 (0.04)	0.69 (0.06)	0.34 (0.05)	0.32 (0.07)	0.28 (0.07)
18	0.38 (0.08)	0.31 (0.08)	0.28 (0.10)	1.42 (0.08)	1.24 (0.09)	1.43 (0.12)	0.26 (0.05)	0.25 (0.07)	0.20 (0.07)
19	0.09 (0.01)	0.09 (0.02)	0.08 (0.02)	0.09 (0.01)	0.09 (0.01)	0.08 (0.02)	1.00 (0.04)	1.00 (0.06)	1.00 (0.09)
20	0.14 (0.02)	0.07 (0.01)	0.15 (0.04)	0.22 (0.02)	0.10 (0.01)	0.32 (0.03)	0.64 (0.06)	0.63 (0.08)	0.49 (0.10)
21	0.02 (0.01)	0.01 (0.00)	0.02 (0.01)	0.12 (0.01)	0.10 (0.01)	0.13 (0.01)	0.15 (0.04)	0.07 (0.04)	0.18 (0.06)
22	0.10 (0.01)	0.07 (0.01)	0.07 (0.01)	0.10 (0.01)	0.07 (0.01)	0.08 (0.01)	1.00 (0.01)	1.00 (0.02)	0.84 (0.05)
23	0.01 (0.01)	0.01 (0.01)	0.00 (0.01)	0.47 (0.03)	0.35 (0.03)	0.61 (0.05)	0.01 (0.02)	0.04 (0.03)	0.00 (0.02)
24	1.88 (0.22)	0.51 (0.12)	2.28 (0.34)	2.71 (0.18)	1.65 (0.13)	2.94 (0.27)	0.69 (0.05)	0.31 (0.07)	0.78 (0.07)
25	2.13 (0.23)	0.79 (0.15)	2.63 (0.38)	2.90 (0.19)	1.60 (0.12)	3.35 (0.30)	0.74 (0.04)	0.49 (0.07)	0.78 (0.06)
26	4.96 (0.39)	0.33 (0.05)	6.95 (0.81)	5.18 (0.34)	0.51 (0.04)	7.42 (0.67)	0.96 (0.02)	0.65 (0.07)	0.94 (0.04)
27	0.01 (0.00)	0.02 (0.00)	NA	0.01 (0.00)	0.02 (0.00)	NA	0.91 (0.02)	0.99 (0.03)	NA
28	1.79 (0.00)	NA	NA	1.79 (0.00)	NA	NA	1.00 (0.00)	NA	NA

Supplementary Table 2. GWAS results from spring and winter barley combined dataset. For each GWAS peak, the quality check (QC) information is provided. QC class A means the peak is represented by two or more markers with significance above FDR of 0.05. QC class B means the peak is represented by one marker above FDR of 0.05 and two or more markers above FDR of 0.10. r^2 is not available for peaks that were detected in the second GWAS (similar to the initial GWAS but with the most significant marker as fixed effect). Since the marker data were coded as -1,0,1 where 1 is the minor allele in the initial marker data with 809 varieties, the effects here are defined with reference to minor allele. C: combined, S: spring, W: winter.

Trait	GWAS peak									Minor allele frequency		
	Marker	Chr	Pos (Mb)	Effect	$-\log_{10}p$	r^2	QC	Major	Minor	C	S	W
1	JHI-Hv50k-2016-250700	4H	525069787	1.15	96.02	0.49	A	G	T	0.19	0.02	0.37
2	JHI-Hv50k-2016-204917	3H	631826807	-0.49	6.40	0.07	B	T	C	0.49	0.05	0.98
3	JHI-Hv50k-2016-36927	1H	473266326	-0.06	4.92	NA	A	T	C	0.16	0.01	0.32
3	JHI-Hv50k-2016-269336	4H	631675144	0.84	116.93	0.59	A	G	C	0.44	0.00	0.91
4	JHI-Hv50k-2016-110539	2H	676764470	-1.01	42.77	0.28	A	G	A	0.25	0.00	0.53
4	JHI-Hv50k-2016-468714	7H	73547899	-0.36	10.67	NA	A	A	G	0.10	0.02	0.19
8	JHI-Hv50k-2016-110406	2H	675755404	-1.24	55.97	0.35	A	A	G	0.25	0.00	0.53
8	JHI-Hv50k-2016-468714	7H	73547899	-0.37	11.01	NA	A	A	G	0.10	0.02	0.19
9	JHI-Hv50k-2016-343	1H	291116	-0.37	6.23	0.04	B	A	G	0.07	0.07	0.06
9	JHI-Hv50k-2016-62012	2H	6179110	-0.23	6.98	0.04	B	T	A	0.43	0.52	0.34
11	SCRI_RS_168399	4H	608432009	-0.22	5.93	0.05	A	A	C	0.45	0.49	0.41
12	JHI-Hv50k-2016-109271	2H	663877140	0.16	6.05	0.05	A	T	G	0.30	0.24	0.37
12	JHI-Hv50k-2016-326756	5H	579729027	-0.18	6.11	0.05	B	G	A	0.42	0.00	0.89
13	JHI-Hv50k-2016-180055	3H	437235827	0.65	5.56	0.03	A	G	C	0.05	0.00	0.11
19	JHI-Hv50k-2016-107749	2H	652418960	-1.00	146.59	0.97	A	G	A	0.29	0.24	0.34
20	JHI-Hv50k-2016-29039	1H	404915672	-0.60	11.39	0.09	A	T	C	0.30	0.02	0.60
20	JHI-Hv50k-2016-108431	2H	655814689	-0.99	19.19	0.16	A	G	A	0.22	0.20	0.25
20	JHI-Hv50k-2016-211623	3H	659544025	-0.29	4.12	0.03	A	C	T	0.16	0.01	0.32
20	JHI-Hv50k-2016-308573	5H	488462871	-0.25	4.37	NA	A	T	C	0.06	0.02	0.11
21	JHI-Hv50k-2016-461364	7H	47563401	0.46	7.93	0.05	A	A	C	0.08	0.07	0.08
22	JHI-Hv50k-2016-314851	5H	542495869	-0.38	24.72	0.15	A	G	A	0.36	0.52	0.18
23	JHI-Hv50k-2016-498733	7H	612520748	-0.45	6.71	0.04	A	C	T	0.06	0.10	0.02
24	JHI-Hv50k-2016-110449	2H	676195259	-0.70	44.08	0.27	A	G	A	0.34	0.14	0.56
24	JHI-Hv50k-2016-468636	7H	72974093	-0.27	8.46	NA	A	T	C	0.22	0.03	0.43
25	JHI-Hv50k-2016-104432	2H	638369307	0.82	57.54	0.32	A	A	G	0.17	0.09	0.26
26	JHI-Hv50k-2016-367876	6H	330928	1.33	152.61	0.64	A	G	A	0.14	0.00	0.29
27	JHI-Hv50k-2016-128079	2H	724707499	-1.00	24.45	0.16	A	T	A	0.48	0.05	0.95
28	JHI-Hv50k-2016-41782	1H	511924294	-0.51	47.74	0.26	A	C	G	0.48	0.00	1.00
28	JHI-Hv50k-2016-275542	4H	643677857	-0.63	63.95	0.34	A	T	C	0.48	0.00	1.00
28	JHI-Hv50k-2016-323150	5H	571032022	-0.34	38.29	0.22	A	G	A	0.46	0.00	0.98

Supplementary Table 3. GWAS results from spring barley only dataset. For each GWAS peak, the quality check (QC) information is provided. QC class A means the peak is represented by two or more markers with significance above FDR of 0.05. QC class B means the peak is represented by one marker above FDR of 0.05 and two or more markers above FDR of 0.10. Since the marker data were coded as -1,0,1 where 1 is the minor allele in the initial marker data with 809 varieties, the effects here are defined with reference to minor allele. C: combined, S: spring, W: winter.

Trait	GWAS peak									Minor allele frequency		
	Marker	Chr	Pos (Mb)	Effect	$-log_{10}p$	r^2	QC	Major	Minor	C	S	W
1	JHI-Hv50k-2016-250940	4H	527125017	1.20	31.84	0.33	A	G	T	0.21	0.06	0.37
8	JHI-Hv50k-2016-416214	6H	536069651	0.51	6.75	0.12	B	A	C	0.30	0.16	0.45
9	SCRI_RS_204276	1H	248411	-0.70	8.80	0.10	A	C	T	0.04	0.06	0.02
15	JHI-Hv50k-2016-263040	4H	608379093	-0.25	4.76	0.07	A	T	C	0.45	0.49	0.41
19	JHI-Hv50k-2016-107749	2H	652418960	-0.99	98.03	0.97	A	G	A	0.29	0.24	0.34
20	JHI-Hv50k-2016-29941	1H	414058556	-0.57	4.53	0.07	A	C	G	0.31	0.06	0.58
20	JHI-Hv50k-2016-108561	2H	656596131	-0.75	8.01	0.12	A	C	T	0.23	0.22	0.25
22	JHI-Hv50k-2016-316002	5H	546260097	-1.04	194.19	0.91	A	G	C	0.22	0.34	0.10
23	JHI-Hv50k-2016-498708	7H	612518575	-0.43	4.55	0.05	A	T	C	0.05	0.10	0.00
24	JHI-Hv50k-2016-110405	2H	675754697	-0.68	19.42	0.25	A	A	G	0.36	0.18	0.57
25	JHI-Hv50k-2016-104388	2H	638193925	1.31	44.49	0.43	A	C	T	0.05	0.09	0.00
27	JHI-Hv50k-2016-128079	2H	724707499	-1.31	27.93	0.30	A	T	A	0.48	0.05	0.95

Supplementary Table 4. GWAS results from winter barley only dataset. For each GWAS peak, the quality check (QC) information is provided. QC class A means the peak is represented by two or more markers with significance above FDR of 0.05. QC class B means the peak is represented by one marker above FDR of 0.05 and two or more markers above FDR of 0.10. r^2 is not available for peaks that were detected in second GWAS (similar to the initial GWAS but with the most significant marker as fixed effect). Since the marker data were coded as -1,0,1 where 1 is the minor allele in the initial marker data with 809 varieties, the effects here are defined with reference to minor allele. C: combined, S: spring, W: winter.

Trait	GWAS peak									Minor allele frequency		
	Marker	Chr	Pos (Mb)	Effect	$-\log_{10}p$	r^2	QC	Major	Minor	C	S	W
1	JHI-Hv50k-2016-251355	4H	531530594	1.00	59.89	0.59	A	T	C	0.32	0.27	0.37
3	JHI-Hv50k-2016-269297	4H	631635187	1.49	61.39	0.59	A	G	C	0.44	0.00	0.91
4	JHI-Hv50k-2016-110120	2H	674233412	-0.78	26.64	0.43	A	A	G	0.26	0.02	0.53
8	JHI-Hv50k-2016-110120	2H	674233412	-0.94	40.39	0.56	A	A	G	0.26	0.02	0.53
8	JHI-Hv50k-2016-468636	7H	72974093	-0.18	5.42	NA	A	T	C	0.22	0.03	0.43
9	JHI-Hv50k-2016-343	1H	291116	-0.82	10.49	0.13	A	A	G	0.07	0.07	0.06
12	JHI-Hv50k-2016-109317	2H	664191672	0.18	5.52	0.07	A	A	T	0.31	0.25	0.37
19	JHI-Hv50k-2016-108296	2H	655509717	-0.99	21.40	0.80	A	G	T	0.24	0.24	0.25
20	JHI-Hv50k-2016-29031	1H	404914383	-0.55	7.33	0.13	A	A	G	0.30	0.02	0.60
20	JHI-Hv50k-2016-107816	2H	653415012	-0.87	9.20	0.17	A	T	C	0.30	0.24	0.37
21	JHI-Hv50k-2016-460614	7H	42684996	1.29	14.60	0.22	A	A	T	0.08	0.10	0.06
22	JHI-Hv50k-2016-316000	5H	546259246	-1.43	64.16	0.59	A	C	G	0.22	0.34	0.10
24	JHI-Hv50k-2016-110120	2H	674233412	-0.86	33.11	0.39	A	A	G	0.26	0.02	0.53
24	JHI-Hv50k-2016-468636	7H	72974093	-0.23	7.44	NA	A	T	C	0.22	0.03	0.43
25	JHI-Hv50k-2016-105043	2H	640060225	0.84	26.69	0.38	A	T	A	0.14	0.03	0.26
26	JHI-Hv50k-2016-368372	6H	1005358	1.04	102.85	0.79	A	A	C	0.20	0.10	0.30

Supplementary Table 5. Summary of Manhattan distances from varying number of random markers.

Number of marker	Manhattan distance	
	Mean	Variance
1	0.912	0.948
2	0.951	0.629
3	0.465	0.251
4	0.727	0.264
5	0.813	0.205
6	0.758	0.156
8	0.673	0.104
10	0.804	0.110
13	0.510	0.077
16	0.595	0.066
20	0.672	0.094
25	0.472	0.032
32	0.514	0.031
40	0.615	0.045
50	0.529	0.027
63	0.608	0.033
79	0.588	0.030
100	0.583	0.024
126	0.555	0.023
158	0.618	0.025
200	0.593	0.020
251	0.637	0.031
316	0.609	0.024
398	0.580	0.026
501	0.607	0.023
631	0.603	0.020
794	0.605	0.023
1000	0.606	0.021
1259	0.604	0.025
1585	0.611	0.023
1995	0.601	0.020
2512	0.609	0.021
3162	0.605	0.022
3981	0.612	0.023
5012	0.611	0.022
6310	0.603	0.023
7943	0.602	0.022
10000	0.603	0.022
12589	0.605	0.021
15849	0.605	0.022
19953	0.602	0.021
25119	0.605	0.022
31623	0.603	0.022
40065	0.604	0.022

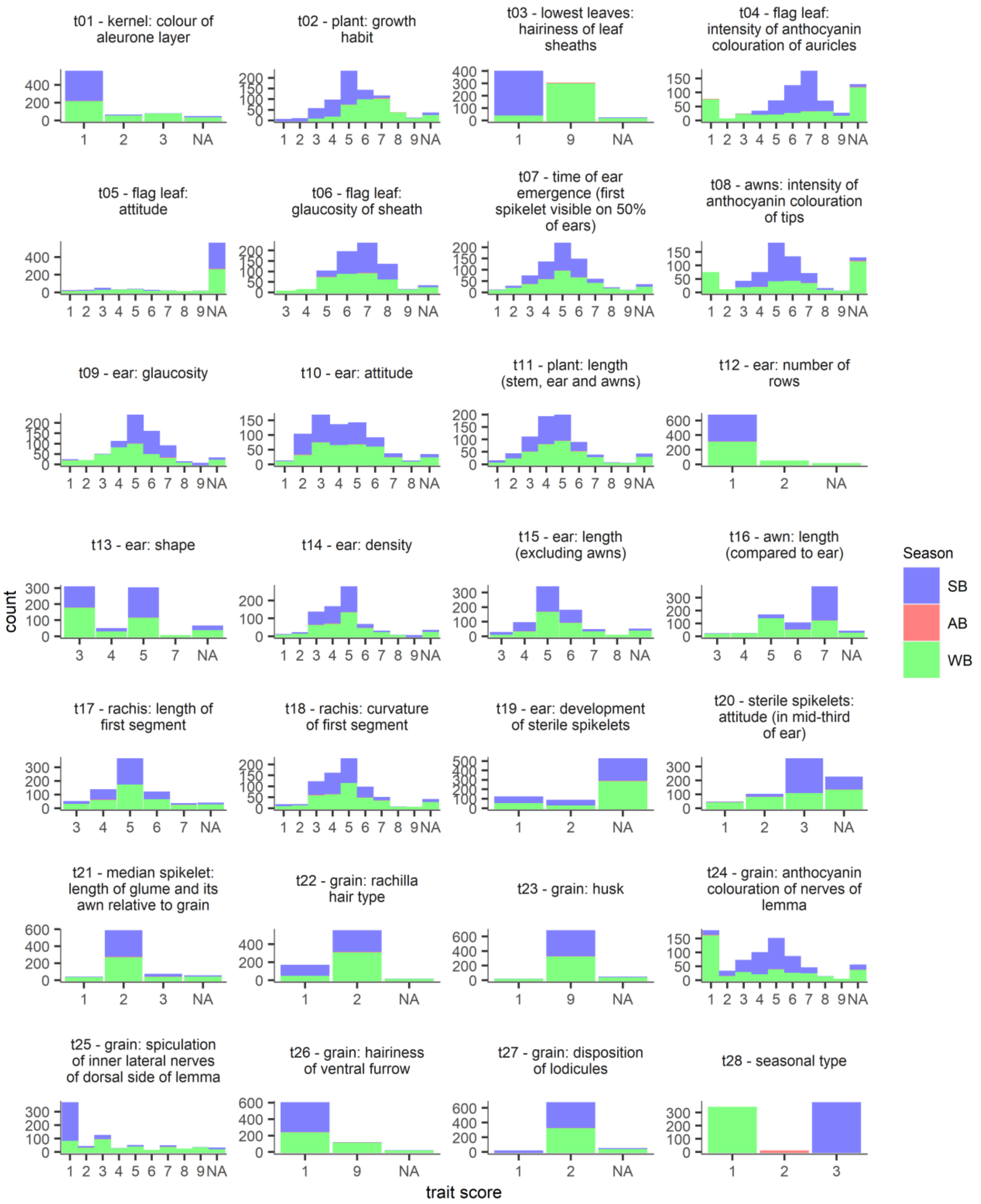

Supplementary Figure 1. Distributions of DUS trait scores. Within each DUS trait, the bars are filled according to seasonal type. SB: spring barley, AB: alternative barley, WB: winter barley.

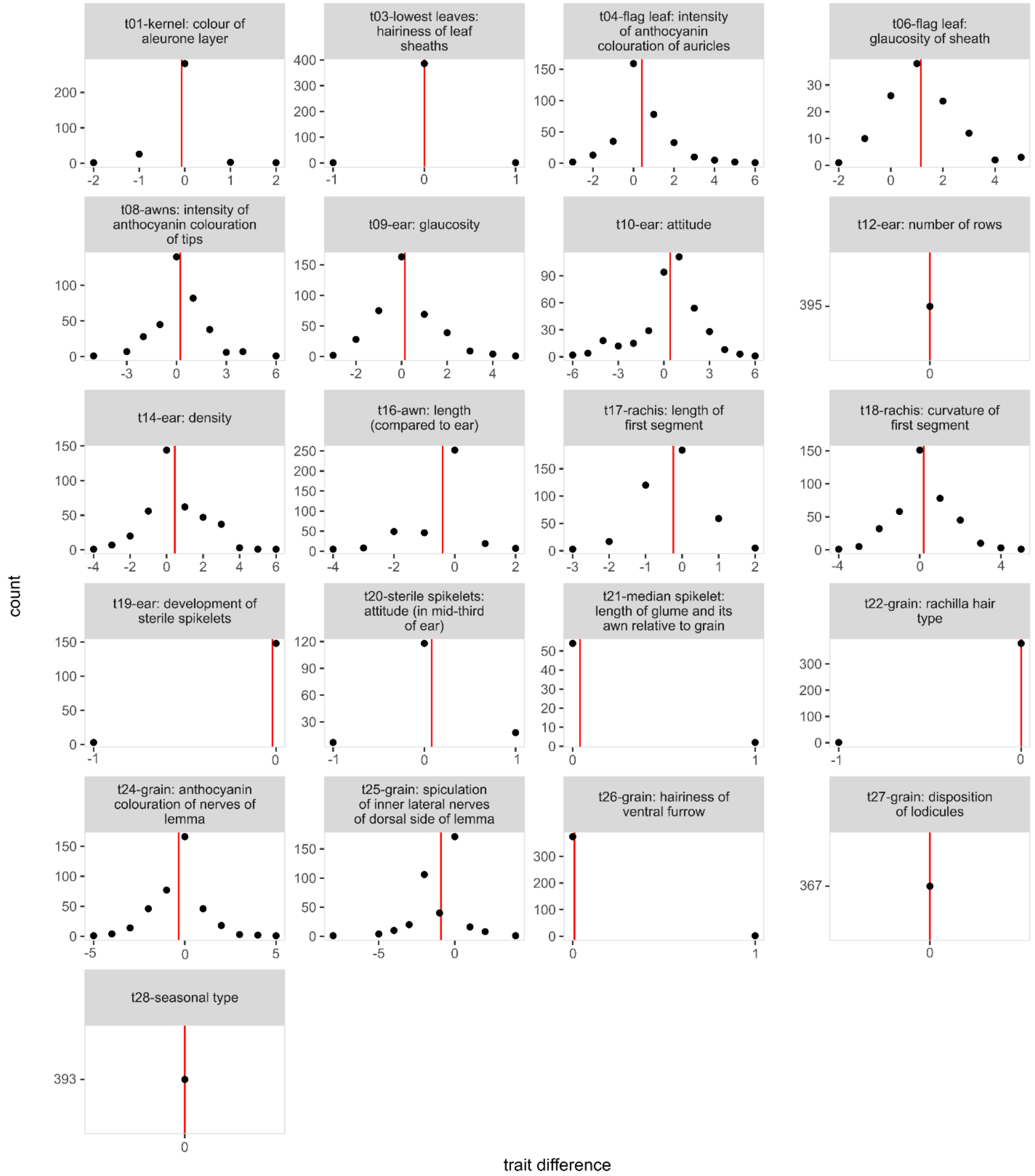

Supplementary Figure 2. Distributions of DUS trait score differences between NIAB and SASA. DUS trait score differences are calculated as trait (NIAB) - trait (SASA). Negative differences mean that NIAB trait score is lower than SASA trait score, and vice versa. There is no variety in common between the NIAB and SASA datasets for 7 traits, and thus no comparison is available for these traits. The trait names are shown above each plot and the means of differences are shown as vertical red lines.

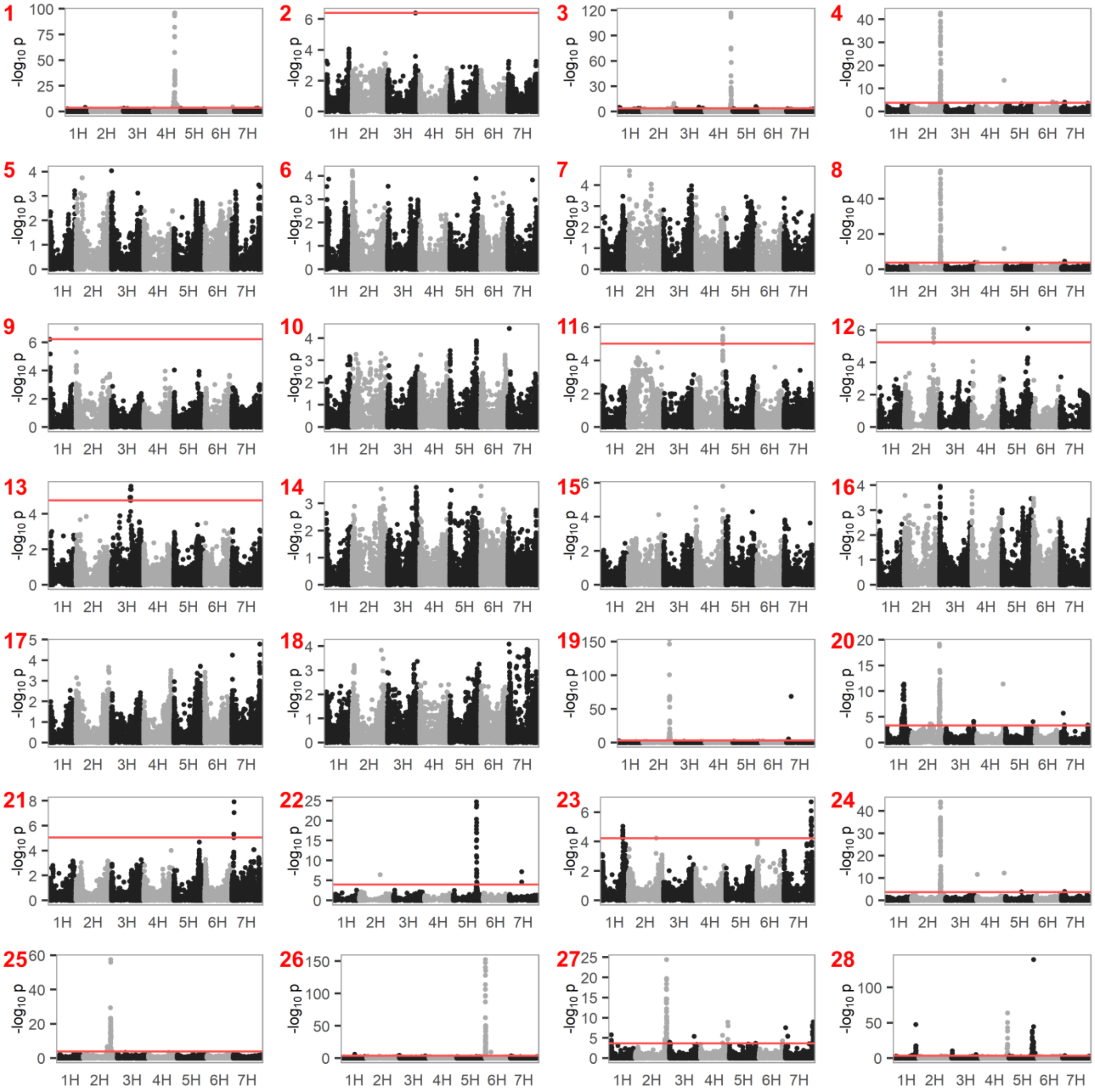

Supplementary Figure 3. Manhattan plots of GWAS results from spring and winter barley combined datasets (n=710). Trait numbers are annotated at the top left corner of each Manhattan plot. Red horizontal line represents FDR = 0.05. If absent no significant marker was identified.

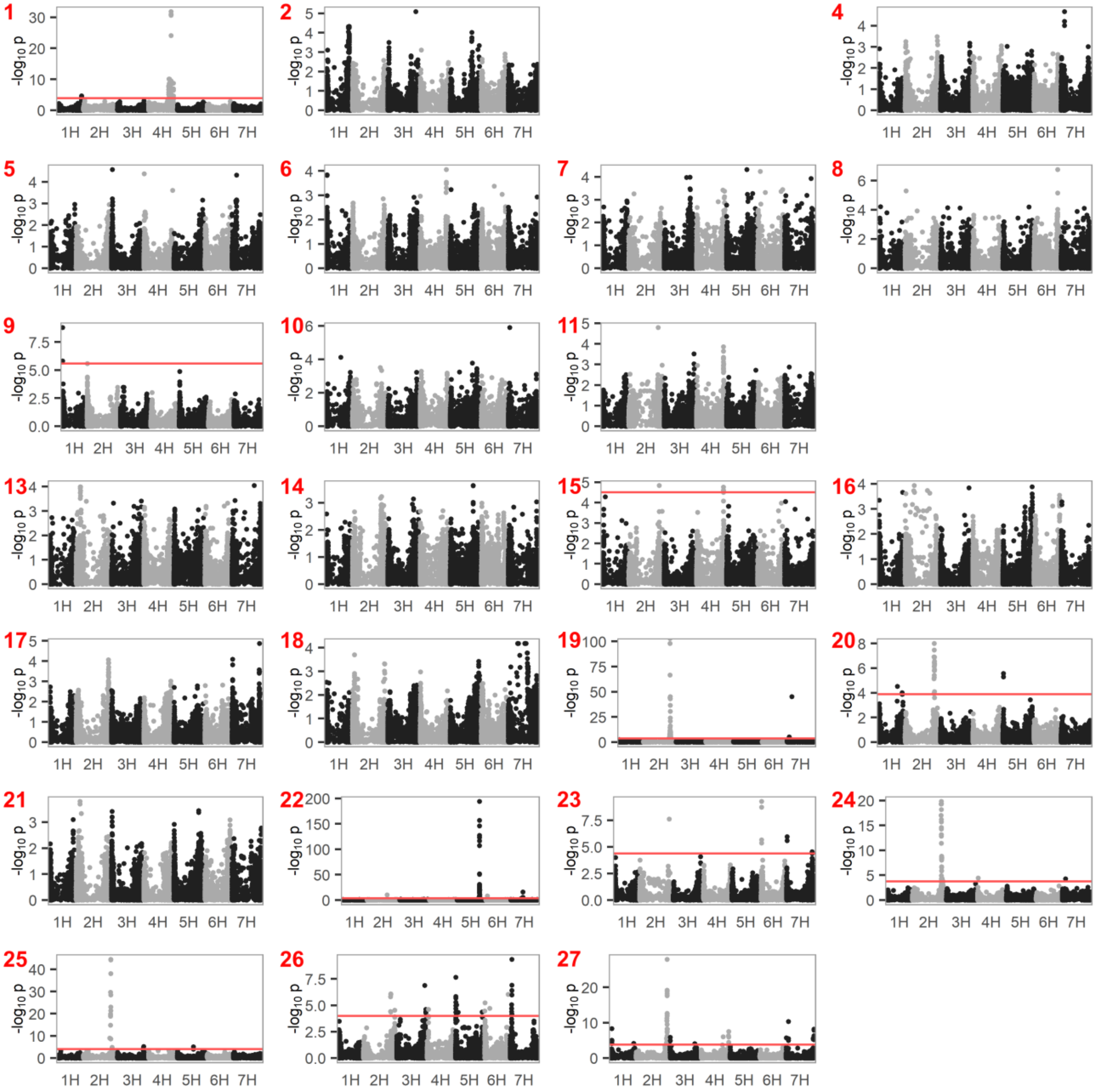

Supplementary Figure 4. Manhattan plots of GWAS results from spring barley only dataset (n=370). Trait numbers are annotated at the top left corner of each Manhattan plot. Red horizontal line represents FDR = 0.05. If absent no significant marker was identified. Plots for trait 3, 12 and 28 are missing here because these traits are not segregating in the spring barley dataset.

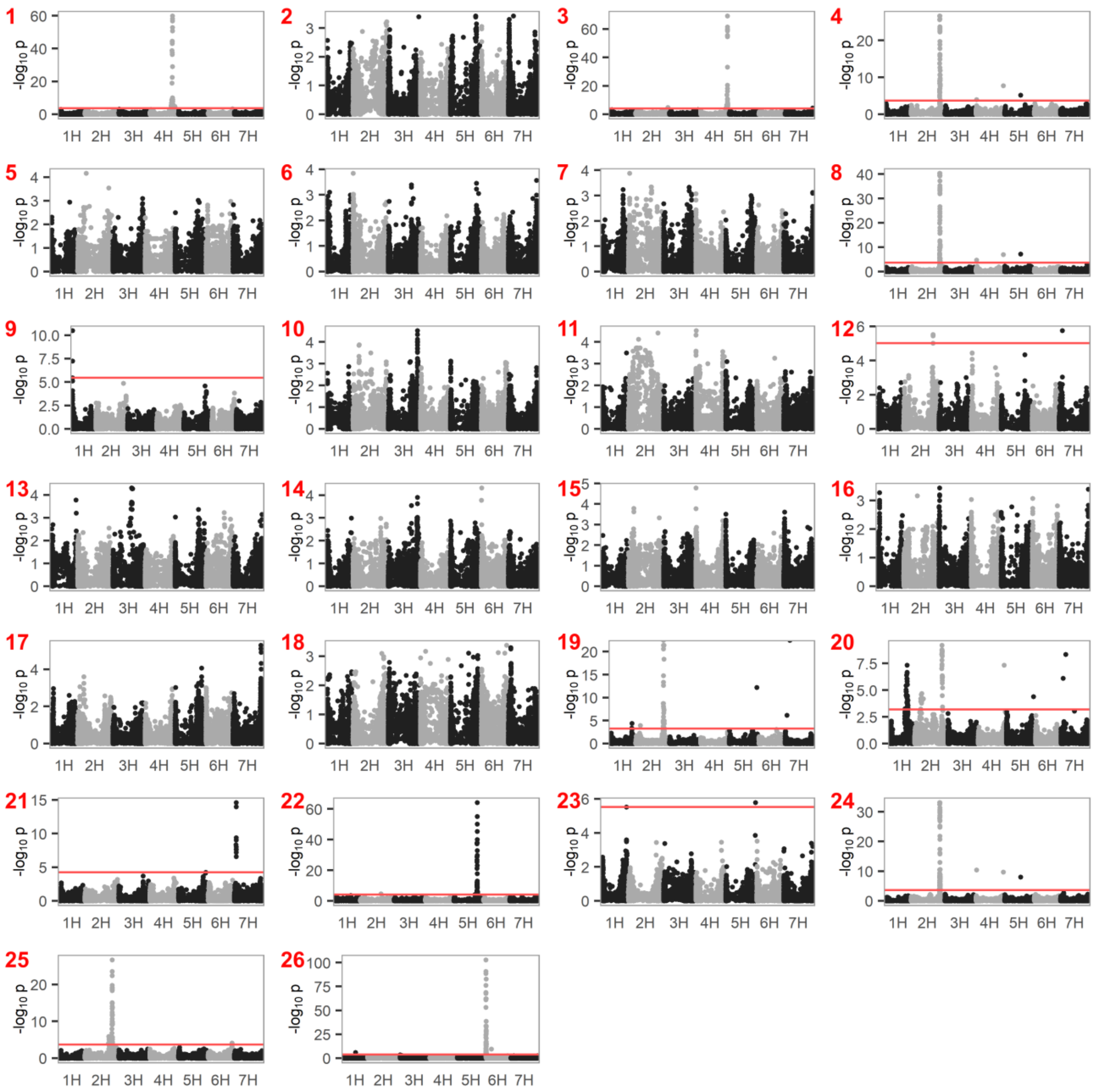

Supplementary Figure 5. Manhattan plots of GWAS results from winter barley only dataset (n=335). Trait numbers are annotated at the top left corner of each Manhattan plot. Red horizontal line represents FDR = 0.05. If absent no significant marker was identified. Plots for trait 27 and 28 are missing here because these traits are not segregating in the winter barley dataset.
