# Supplementary material for: Overcoming barriers to the registration of new plant varieties under the DUS system
Source: Commun Biol. 2021 Mar 8;4:302. doi: 10.1038/s42003-021-01840-9 (PMC7940638; doi:10.1038/s42003-021-01840-9)
Supplement: Supplementary file 3 — Description of Additional Supplementary Files [file 42003_2021_1840_MOESM3_ESM.pdf]

Description of Additional Supplementary Files

File Name: Supplementary Data 1

Description: DUS trait scoring system for UK barley

File Name: Supplementary Data 2

Description: Comparison of simulated progeny with small marker set.

File Name: Supplementary Data 3

Description: DUS trait data from NIAB

File Name: Supplementary Data 4

Description: DUS trait data from SASA

File Name: Supplementary Data 5

Description: Dry matter yield data.
