# Supplementary material for: Overcoming barriers to the registration of new plant varieties under the DUS system
Source: Commun Biol. 2021 Mar 8;4:302. doi: 10.1038/s42003-021-01840-9 (PMC7940638; doi:10.1038/s42003-021-01840-9)
Supplement: Supplementary file 9 — Reporting summary [file 42003_2021_1840_MOESM9_ESM.pdf]

nature research

Corresponding author(s): Ian Mackay

Last updated by author(s): Jan 5, 2021

Reporting Summary

Nature Research wishes to improve the reproducibility of the work that we publish. This form provides structure for consistency and transparency in reporting. For further information on Nature Research policies, see our Editorial Policies and the Editorial Policy Checklist.

Statistics

For all statistical analyses, confirm that the following items are present in the figure legend, table legend, main text, or Methods section.

n/a	Confirmed	
☐	☒	The exact sample size (*n*) for each experimental group/condition, given as a discrete number and unit of measurement
☒	☐	A statement on whether measurements were taken from distinct samples or whether the same sample was measured repeatedly
☐	☒	The statistical test(s) used AND whether they are one- or two-sided<br>*Only common tests should be described solely by name; describe more complex techniques in the Methods section.*
☐	☒	A description of all covariates tested
☐	☒	A description of any assumptions or corrections, such as tests of normality and adjustment for multiple comparisons
☐	☒	A full description of the statistical parameters including central tendency (e.g. means) or other basic estimates (e.g. regression coefficient) AND variation (e.g. standard deviation) or associated estimates of uncertainty (e.g. confidence intervals)
☐	☒	For null hypothesis testing, the test statistic (e.g. *F*, *t*, *r*) with confidence intervals, effect sizes, degrees of freedom and *P* value noted<br>*Give P values as exact values whenever suitable.*
☒	☐	For Bayesian analysis, information on the choice of priors and Markov chain Monte Carlo settings
☒	☐	For hierarchical and complex designs, identification of the appropriate level for tests and full reporting of outcomes
☒	☐	Estimates of effect sizes (e.g. Cohen's *d*, Pearson's *r*), indicating how they were calculated

Our web collection on statistics for biologists contains articles on many of the points above.

Software and code

Policy information about availability of computer code

Data collection	None.
Data analysis	The custom R scripts for all analyses are available at https://github.com/cjyang-sruc/DUS. R version 4.0.2 was used and the associated R packages include reshape2_1.4.4, ggplot2_3.3.2, ggrepel_0.8.2, lme4_1.1-23, emmeans_1.5.2-1, sommer_4.1.1, AlphaSimR_0.12.2 and qvalue_2.20.0.

For manuscripts utilizing custom algorithms or software that are central to the research but not yet described in published literature, software must be made available to editors and reviewers. We strongly encourage code deposition in a community repository (e.g. GitHub). See the Nature Research guidelines for submitting code & software for further information.

Data

Policy information about availability of data

All manuscripts must include a data availability statement. This statement should provide the following information, where applicable:

- Accession codes, unique identifiers, or web links for publicly available datasets
- A list of figures that have associated raw data
- A description of any restrictions on data availability

The compiled DUS trait data from NIAB and SASA and BLUEs for dry matter yield are available in Supplementary File 1. The IMPROMALT marker data is available at http://www.barleyhub.org/projects/impromalt/ subject to permission from the James Hutton Institute. All source file website links have been archived at https://web.archive.org/.

Field-specific reporting

Please select the one below that is the best fit for your research. If you are not sure, read the appropriate sections before making your selection.

- [x] Life sciences
- [ ] Behavioural & social sciences
- [ ] Ecological, evolutionary & environmental sciences

For a reference copy of the document with all sections, see nature.com/documents/nr-reporting-summary-flat.pdf

Life sciences study design

All studies must disclose on these points even when the disclosure is negative.

Sample size	Sample size were chosen based on available genotypic marker data from the IMPROMALT project, which includes 805 spring, winter and alternative barley varieties that are registered in the UK crop variety registration system over the years.
Data exclusions	Samples without DUS trait or yield data were excluded from their respective analyses. The lack of phenotypic data for some samples is because the DUS trait and yield data were sourced independently from the marker data.
Replication	The DUS trait data is determined over two years of field trials while the yield data is determined over variable number of years of field trials. We do not have control over the replications here as they have been pre-determined by the running organisations.
Randomization	Groups were determined by year of variety release) and seasonal type. These covariates were included in full analyses on all samples.
Blinding	Blinding is not relevant in this study.

Reporting for specific materials, systems and methods

We require information from authors about some types of materials, experimental systems and methods used in many studies. Here, indicate whether each material, system or method listed is relevant to your study. If you are not sure if a list item applies to your research, read the appropriate section before selecting a response.

Materials & experimental systems

n/a	Involved in the study	
x		Antibodies
x		Eukaryotic cell lines
x		Palaeontology and archaeology
x		Animals and other organisms
x		Human research participants
x		Clinical data
x		Dual use research of concern

Methods

n/a	Involved in the study	
x		ChIP-seq
x		Flow cytometry
x		MRI-based neuroimaging
